# Which East Asian herbal medicines can decrease viral infections?

**DOI:** 10.1007/s11101-021-09756-2

**Published:** 2021-08-27

**Authors:** Kenny Kuchta, Silke Cameron, Minwon Lee, Shao-Qing Cai, Yukihiro Shoyama

**Affiliations:** 1 Forschungsstelle Für Fernöstliche Medizin, Department of Vegetation Analysis and Phytodiversity, Albrecht Von Haller Institute of Plant Sciences, Georg August University, Göttingen, Germany; 2 Clinic for Gastroenterology and Gastrointestinal Oncology, University Medical Center Göttingen, 37075 Göttingen, Germany; 3 Laboratory of Pharmacognosy and Natural Product-Based Medicine, College of Pharmacy, Chung-Ang University, Seoul, 156-756 Korea; 4 International Cooperative Center for Researches of Medicinal Resources, Peking University Health Center, Peking University, Haidian District, Beijing, 100191 China; 5 Faculty of Pharmacy, Nagasaki International University, 2825-7, Sasebo, Nagasaki 859-3298 Japan

**Keywords:** COVID-19, Influenza virus, *Andrographis paniculata*, Kampo, Maoto

## Abstract

Whilst Western research for the COVID-19 crisis focuses on vaccination, in East Asia traditional herbal prescriptions are studied for SARS-CoV2 therapy. In Japan, Maoto (Ephedrae herba 4 g, Armeniacae semen 4 g, Cinnamomi cortex 3 g, and Glycyrrhizae radix 2 g, JPXVII) is used based on clinical evidence for its effect on early phase influenza (also caused by RNA viruses) comparable to that of oseltamivir. The Health Ministry of Thailand has approved* Andrographis paniculata* (Jap. Senshinren) extracts for treatment of COVID-19. Its combination (4 g) with Maoto, Maoto-ka-senshinren, seems most promising for the treatment of viral pandemics. In China, the official guideline for COVID-19 treatment contains TCM medications with antiviral, as well as immunmodulatory and anti-inflammatory effects such as: Qing-Fei-Pai-Du-Tang (Jap. Seihai-haidokuto) contains 21 drugs; Shufeng Jiedu Jiaonang (Bupleuri radix 8 g, Forsythiae fructus 8 g, Glycyrrhizae radix 4 g, Isatidis radix 8 g, Patriniae herba 8 g, Phragmitis rhizoma 6 g, Polygoni cuspidati rhizoma 10 g, Verbenae herba 8 g); Fufang Yuxingcao Heiji (Forsythiae fructus 0.6 g, Houttuyniae herba 6 g, Isatidis radix 1.5 g, Lonicerae flos 0.6 g, Scutellariae radix 1.5 g) first gained prominence during the 2002 SARS epidemic. With no Western medicine available, the following overview discusses efficacy and mechanisms in view of viral entry and replication of different East Asian herbal remedies for COVID-19 treatment.

## Introduction

Anti-viral activity has been reported from numerous medicinal plant extracts and preparations. For example, Mousa (2017) reviewed the anti-influenza activity of several medicinal plants such as *Glycyrrhiza uralensis*, *Panax ginseng*, *Camellia sinensis*, or *Diospyros kaki*. In our own research work, we were able to demonstrate that the pharmaceutical oleoresin Labdanum of *Cistus creticus* exerts pronounced in vitro anti-dengue virus activity (Kuchta et al. 2019).

The most probable targets of anti-viral natural products are those related to the replication cycle of viruses, which depends on several steps: Their recognition of the host cell, formation of the endosome, release of virus RNA within the cytoplasm, RNA replication and translation. The new endosome can then release the virus by exocytosis. All these steps can be targeted by entry-inhibitors, fusion inhibitors, RNA polymerase inhibitors, protease- and release inhibitors.

The commonly used Oseltamivir is a neuraminidase inhibitor, i.e. a competitive inhibitor of the viral neuraminidase enzyme. Inhibition of the enzyme prevents cleaving of the virus from the host cell, and thus prevents the spreading of the virus. However, several resistance mechanisms are known. Current studies suggest that it is not effective in the treatment of the new COVID-19 pandemic. However, East Asian herbal medicines have shown anti-viral activity in the past and in the current reports on corona viruses.

For the current overview, Kampo prescriptions commonly recommended in Japan were assessed together with related herbal medications from China, Korea, and Thailand.

Although the past year has seen a flood of papers on Chinese medicines for the treatment of COVID-19, most of the prescriptions discussed therein are either new, previously untested drug combinations and / or hardly available internationally. Thus a selection was done for such prescriptions that are either commonly available on international markets or already established in the practice of Eastern Medicine in Europe. Especially established prescriptions—and simple variations thereof—that can easily be formulated as Single Prescriptions for individual patients from decoction pieces by doctors in the West were included.

## Anti-viral activity of *Andrographis paniculata*

**Fig. 1:**
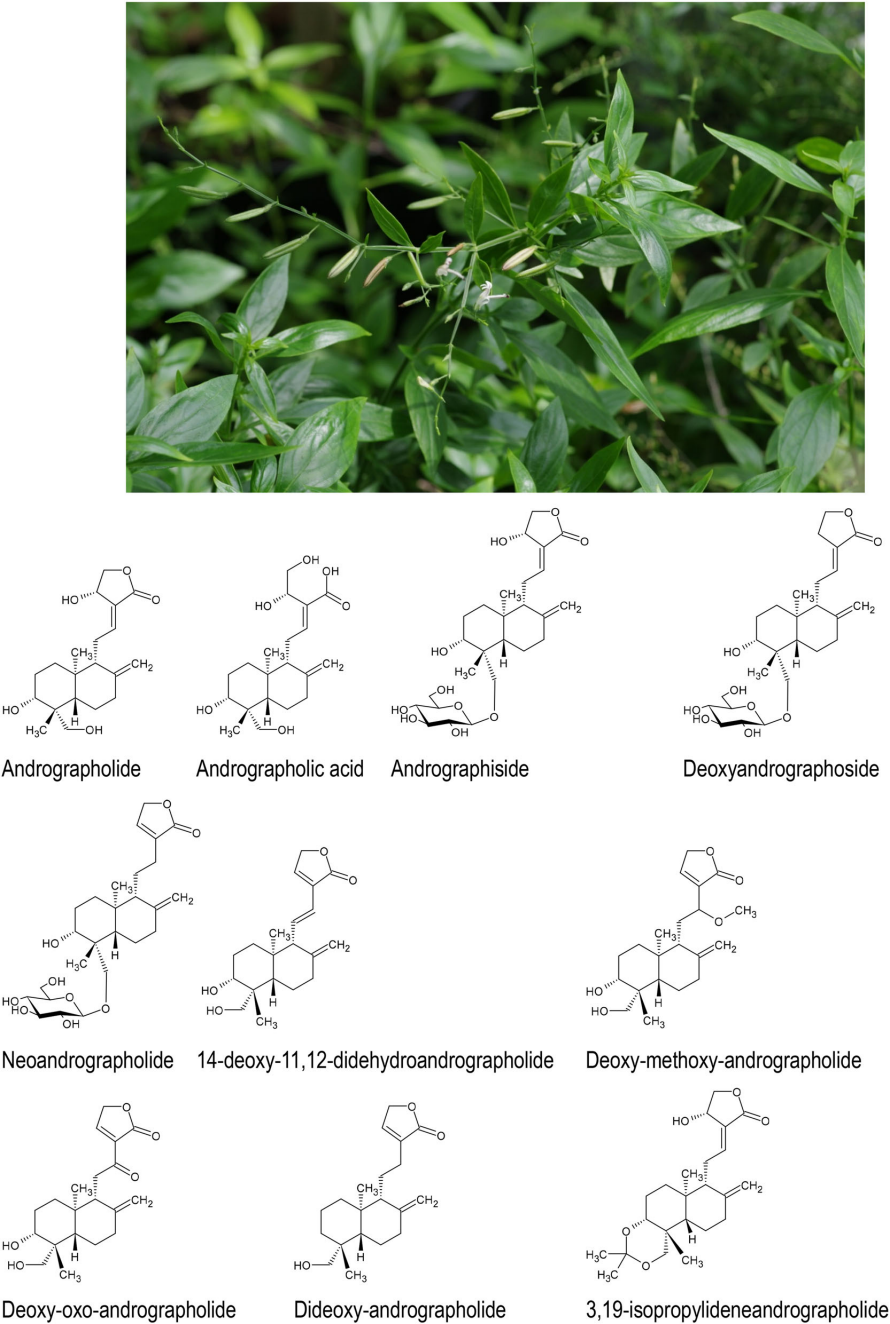
*Andrographis paniculata* (Burm.f.) Nees and some of its active constituents EphedrineNorephedrine Pseudoephedrine D-Norpseudoephedrine L-Methylephedrin L-Ephedrine D-Pseudo-ephedrine

*Andrographis paniculata* (Burm.f.) Nees (Fig. 1) is native to tropical Southeast Asia and traditionally used in Indian Ayurvedic medicine, traditional Thai medicine and traditional Indonesian Jamu medicine (Herrmann 1996) against diarrhea, bacterial dysentery and as a bitter tonic for numerous diseases. Especially in Jamu, *A. paniculata* is also well documented as a traditional treatment for malaria (Herrmann 1996).

In Thai Traditional Medicine, *A. paniculata* (Th. ฟ้าทะลายโจร / Fha talai jone) is one of the most commonly used herbal drugs (Inta et al. 2013) and often administered as a decoction or pill for the prevention of all health problems. It is also used for the treatment of numerous ailments such as fever, cough, sore throat, apthous ulcer, wounds, abscesses, rashes, and as a carminative, for gastritis, pain, diabetes, hypertension, jaundice, and for "detoxification" (Inta et al. 2013).

Later, this medicinal plant was adopted into Traditional Chinese Medicine (TCM) as an antipyretic and against bronchitis, colitis, cystitis and similar inflammatory diseases (Wagner et al. 2011). Most recently, its introduction into the Chinese pharmacopoeia has resulted in its insertion into the newest edition of the European Pharmacopoeia (PhEur 9) and, due to current efforts to integrate more herbal drugs of East Asian Medicine, into the European regulatory framework.

The efficacy and safety of *A. paniculata* containing preparations for prophylaxis and symptomatic treatment of respiratory infections such as the common cold, bronchitis, sinusitis pharyngotonsillitis, urinary tract infections, and acute diarrhea has been supported by clinical studies, as laid out in a recent HMPC Assessment report (EMA/HMPC/320433/2012). Numerous, in the context of COVID-19 especially relevant studies in patients with viral lounge infections were performed in Scandinavia, South America, and India (Hancke et al. 1995; Caceres et al. 1997, 1999; Melchior et al. 1996; Saxena et al. 2010). A meta-analysis of 33 randomized controlled trials showed that *A. paniculata* extracts relieve inflammatory symptoms and shortens the duration of cough, sore throat, and disease duration in comparison with standard care (Hu et al. 2017). Saxena et al. (2010) carried out a randomized double blind placebo controlled clinical evaluation of *A. paniculata* extract in patients with uncomplicated upper respiratory tract infection regarding cough, fever, sputum, nasal mucus, headache, fatigue and sleep disorders resulting in 53% of improvement compared with placebo. When 158 common cold patients took 1.2 g of dried extract of *A. paniculata* for 5 days, symptoms like sleep disorder, nasal juice and sore throat improved. Akbar (2011) examined *A. paniculata* for the prevention and treatment of the common cold. 158 adult patients suffering from common cold used a standardized *A. paniculata* dry extract for 5 days, resulting in a significant decrease of tiredness, sleeplessness, sore throat and nasal secretion.

Ding et al. (2017) demonstrated that treatment of C57BL/6 mice infected with the mouse-adapted H1N1 strain PR8A/PR/8/34 with andrographolide [10 mg/kg], with or without influenza virus entry inhibitor CL-385319 [10 mg/kg] improved body weight, lung function and reduced inflammation. The combination group had the highest survival rate but andrographolide treatment alone improved the survival rate as well as virus loads and inflammatory cytokine expression (Ding et al. 2017). Numerous natural products have been isolated from *A. paniculata* several of which—such as diverse lactones, flavonoids, diterpenes, and especially andrographolides (Fig. 1)—are regarded as contributors to its documented activity against influenza viruses.

All the above indicates that *A. paniculata* can be used for the prevention and treatment of the common cold and even influenza virus infection. It remains to be shown if *A.* *paniculata* is also effective in SARS-COVID-19 infection. However, similar strategies such as protease inhibition and cell modulation of cell surface receptors preventing viral entry have been shown in other viral infections including HIV. Furthermore, *A. paniculata* is well known for its anti-malaria activity (Herrmann 1996). Its activity is quite similar to that of chloroquine—a synthetic anti-malaria compound derivative of the alkaloid quinine that has been proposed for co-medication with *A. paniculata* in this indication (Hafid et al. 2015)—and is currently investigated for the treatment of COVID-19 infection.

Based on these findings and its established status in Thai Traditional Medicine, the Health Ministry Thailand has approved the use of the extract to treat early stages of Covid-19 as a pilot program (The Straits Times. DEC 30, 2020).

As far as the specific mechanism of action of *A. paniculata* against viral infections is concerned, it has been experimentally demonstrated that the andrographolide 14-deoxy-11,12-dehydroandrographolide (DAP) (Fig. 1), a major component of the raw drug with a minimum content of 0.8% of the sum of andrographolide and DAP in the dried drug material according to Ph.Eur., exerts potent anti-influenza A virus activity against A/chicken/Hubei/327/2004 (H5N1), A/duck/Hubei/XN/2007 (H5N1), A/PR/8/34 (H1N1), A/NanChang/08/2010 (H1N1) and A/HuNan/01/2014 (H3N2) in vitro on A549 and MDCK cells and inhibits the replication of the H5N1 influenza virus by preventing the export of the viral ribonucleoprotein complexes from the nucleus (Cai et al. 2015).

For H5N1, DAP exhibited a CC50 (cytotoxic concentration required to reduce cell viability by 50% for uninfected cells determined by CCK-8 assay) and an IC50 (inhibition concentration to reduce the cytopathic effect (CPE) by 50% caused by A/chicken/Hubei/327/2004 (H5N1) in the same order of magnitude as the positive control Ribavirin (Cai et al. 2015).

Andrographolide itself was also shown to contribute to the overall anti-viral activity of *A. paniculata* extracts. In the case of the enterovirus D68 (EV-D68), that has emerged as a significant respiratory pathogen in recent years, it could be demonstrated in an in vitro virus model on human rhabdomyosarcoma RD cells (ATCC, CCL-136) that andrographolide prevents its replication by inhibiting the acidification of virus-containing endocytic vesicles, resulting in a dramatic inhibition of EV-D68 RNA replication (EC50 = 3.45 mM). In comparison, its median cytotoxic, lethal concentration was much higher at 75 mM (Wang et al. 2018).

*A. paniculata* in general and andrographolide in particular have also proven effective against the dengue virus (Paemanee et al. 2019). Here, the human HepG2 liver cell (ATCC Cat No. HB-8065) were infected with DENV 2 and subsequently incubated with andrographolide (50, 100, and 200 μM). A proteomic based approach demonstrated an important role for Glucose regulated protein 78 (GRP78) and the unfolded protein response (UPR) mechanism in mediating the anti-dengue virus activity of andrographolide, which might, in part, explain the broad antiviral activity of andrographolide (Paemanee et al. 2019).

In this context, yet another andrographolide, 3,19-isopropylideneandrographolide (IPAD) was shown to be effective against Herpes Simplex Virus. IPAD (22.50 µM) completely suppressed ICP8 transcription and translation as well as DNA replication and HSV gD protein (Envelope glycoprotein D of the Human herpesvirus 1 (strain 17) (HHV-1) (Human herpes simplex virus 1)) expression in the tested virus strains in a Vero host cell model (Kongyingyoes et al. 2016). This envelope glycoprotein binds to the potential host cell entry receptors like TNFRSF14/HVEM, and NECTIN1 and may trigger fusion with host membrane by recruiting the fusion machinery.

A comparison of human patient data with those from a rat model showed that the pharmacokinetics of andrographolides are similar in both species. They are rapidly and almost completely absorbed (T1/2abs of about 25 min) into the blood (bioavailability = 91%, F = 0.91) after oral administration at a therapeutic dose (20 mg/kg). Andrographolide binds to blood proteins and is distributed in blood and tissues within 1–2 h. The elimination half-time is in the range of 2–7 h (Panossian et al. 2000). A tissue distribution study revealed the highest concentration in kidney, followed by the liver, spleen, and brain, whereas an almost identical concentration was observed in heart and lungs (Bera et al. 2014).

It is however important to note that bioactivity of andrographolides is not limited to the anti-viral effect itself but also affects therapeutically relevant side effects of the infection. E.g. andrographolide was shown to inhibit Influenza A virus induced inflammation in a murine model through NF-κb and JAK-STAT signaling pathway (Ding et al. 2017).

In this context, it is important to note that *A. paniculata* has also been discussed as an "Adaptogen" (Panossian et al. 2021)—a category of natural compounds or herbal extracts that increase adaptability, resilience, and survival of organisms; they increase “the state of nonspecific resistance” of organisms to harmful factors, including bacterial and viral pathogens (Lazarev et al. 1959). In Ayurveda, the plants with traditionally use as adaptogens are referred to as "Rasayana" and are used as rejuvenating and for improving the overall health of anyone undergoing this treatment. It is therefore not surprising that *A. paniculata* is regarded as one of the most important rasayana drugs (Thakur et al. 2014, 2015; Raina et al. 2013). In this context, the chemopreventive effects of *A. paniculata* extracts and Andrographolide were previously demonstrated (Sheeja and Kuttan 2006; Singh et al. 2009).

## Anti-viral activity of Kampo prescriptions such as Maoto

In Japan several Kampo prescriptions like Maoto, Kakkonto (Kurokawa et al. 1996; Okabayashi et al. 2014), Shahakusan (Hokari et al. 2012), Shoseiryuto (Nagai and Yamada 1994, 1998; Nagai et al. 1996; Yamada and Nagai 1998), Daiokanzoto (Watanabe 2018) and Hochuekkito (Dan et al. 2018) (Table 1) have been investigated for their effect against influenza virus infection.

**Table 1:** Anti-viral Kampo prescriptions including their "one-day dose of crude drugs" for decoction according to JPh

Maoto 麻黄湯		Kakkonto 葛根湯		Shahakusan 瀉白散		Shoseiryuto 小青竜湯		Daiokanzoto 大黄甘草湯		Hochuekkito 補中益気湯	
										Angelicae sinensis radix	3 g
Armeniacae semen	4 g										
						Asiasari radix	3 g				
										Astragali radix	4 g
										Atractylodis mac. rhizoma	4 g
										Bupleuri radix	2 g
										Cimicifugae rhizoma	1 g
Cinnamomi cortex	3 g	Cinnamomi cortex	3 g			Cinnamomi cortex	3 g				
										Citri reticulatae pericarpium	2 g
Ephedrae herba	4 g	Ephedrae herba	4 g			Ephedrae herba	3 g				
										Ginseng radix	4 g
Glycyrrhizae radix	2 g	Glycyrrhizae radix	2 g	Glycyrrhizae radix	2 g	Glycyrrhizae radix	3 g	Glycyrrhizae radix	2 g	Glycyrrhizae radix	1 g
		Jujubae fructus	4 g							Jujubae fructus	2 g
				Lycii radicis cortex	4 g						
				Mori radicis cortex	4 g						
		Paeoniae radix	3 g			Paeoniae radix	3 g				
						Pinelliae rhizoma	6 g				
		Puerariae radix	8 g								
								Rhei rhizoma	4 g		
						Schisandrae fructus	3 g				
		Zingiberis rhizoma	1 g			Zingiberis rhizoma	3 g			Zingiberis rhizoma	1 g
				Long-grained rice	2 g						

All the above are traditionally prepared as decoctions (i.e. hot water extracts) according to the legal requirements of the current Japanese Pharmacopoeia (JPXVII, p. 22): "Heat one-day dose of crude drugs with 400–600 ml of water until loss of about half the amount of added water spending more than 30 min, and filter through a cloth while warm."

**Fig. 2:**
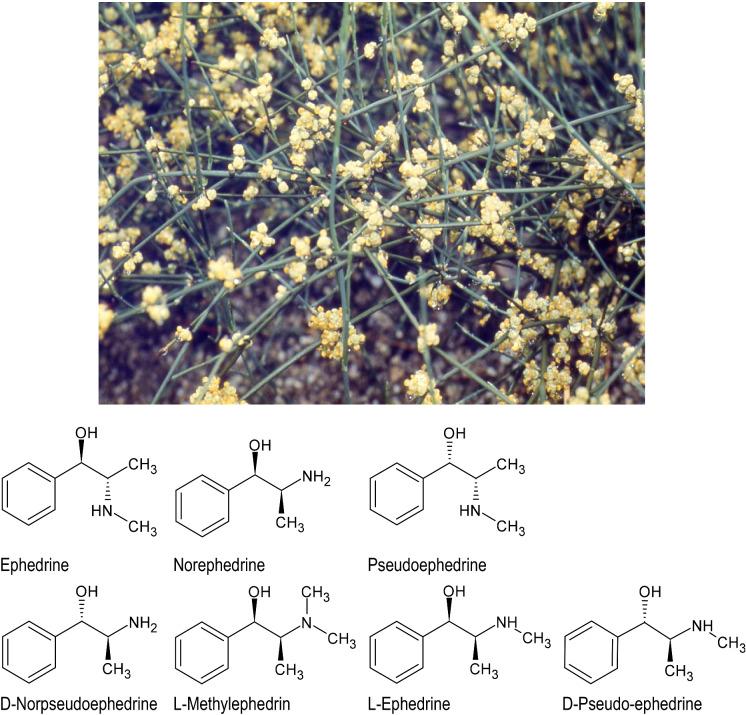
*Ephedra sinica* Stapf and some of its active constituents

Among all examined Kampo prescriptions, the most detailed information was available for Maoto (Ephedrae herba 4 g, Armeniacae semen 4 g, Cinnamomi cortex 3 g, and Glycyrrhizae radix 2 g). This prescription will therefore form the basis for the further discussion in the following paragraphs. Maoto (麻黄湯) was published already during the second century AD in the Shanghan Lun (傷寒論, Jap. Shoukanron)—one of the foundational texts of Ancient Chinese Medicine—under the Chinese name Ma-Huang-Tang, under which it is still used in TCM today. In Korean medicine, which developed from Ancient Chinese Medicine as a third sister system to TCM and Kampo, the identical prescription is referred to as Mahwang-tang (마황탕). Maoto is commonly applied for febrile diseases with symptoms like high fever and cough. Kampo prescriptions like Maoto that contain Ephedrae herba (*Ephedra sinica* Stapf) (Fig. 2) are also referred to as Mao-zai (麻黄剤).

Recently, Kampo clinical trials for influenza virus infection have been reported in Japan. E.g. Nagai et al. (2014) found that orally administered Maoto (0.9 and 1.6 g/kg/day) had significant anti-pyretic activity in influenza virus infected A/J mice after upper respiratory tract infection with the influenza virus A/PR/8/34. Administration of Maoto (0.8 and 1.3 g/kg/day) further significantly decreased the virus titers in both nasal and bronchoalveolar lavage fluids 52 h after infection and increased the anti-influenza virus antibodies IgM, IgA and IgG1 resulting in the binding of the virus (Nagai et al. 2014).

Masui et al. (2017) set up the culturing assay system for A549 cells which were infected with influenza virus A (PR8) in order to determine the virus titers in the culture supernatant, intracellular viral proteins and viral RNA. When the infected cells were treated with 400 µg/ml of a commercial Maoto, the extract significantly reduced the virus titer as well as the production of viral surface proteins such as M2 and neuraminidase (NP), thus preventing viral entry (Masui et al. 2017).

Maoto can also inhibit the uncoating of influenza virus. Furthermore, the inhibition of endosomal acidification by Maoto may prevent the release of the influenza virus through the inhibition of V-ATPase into the cytoplasm (Masui et al. 2017). Maoto is therefore the most favorable Kampo medicine for influenza virus and influenza illness.

Nabeshima et al. (2012) investigated Maoto [2.5 g TID of commercial granules dissolved in warm water for 5 days] for the treatment of seasonal influenza in a randomized clinical trial. 28 influenza patients within 48 h of fever onset were randomly assigned to Maoto (n = 10), Oseltamivir [75 mg BID for 5 days] (n = 8), or Zanamivir [20 mg BID for 5 days] (n = 10) and data collected for their total symptom score from self-reported symptom cards and the duration of fever (> 37.5 °C). No significant between-group differences were found for total symptom score among three groups without severe adverse effects. Nabeshima et al. (2012) thus demonstrated that Maoto affects the early phase of influenza virus infection, with an anti-influenza activity comparable to that of oseltamivir.

**Fig. 3:**
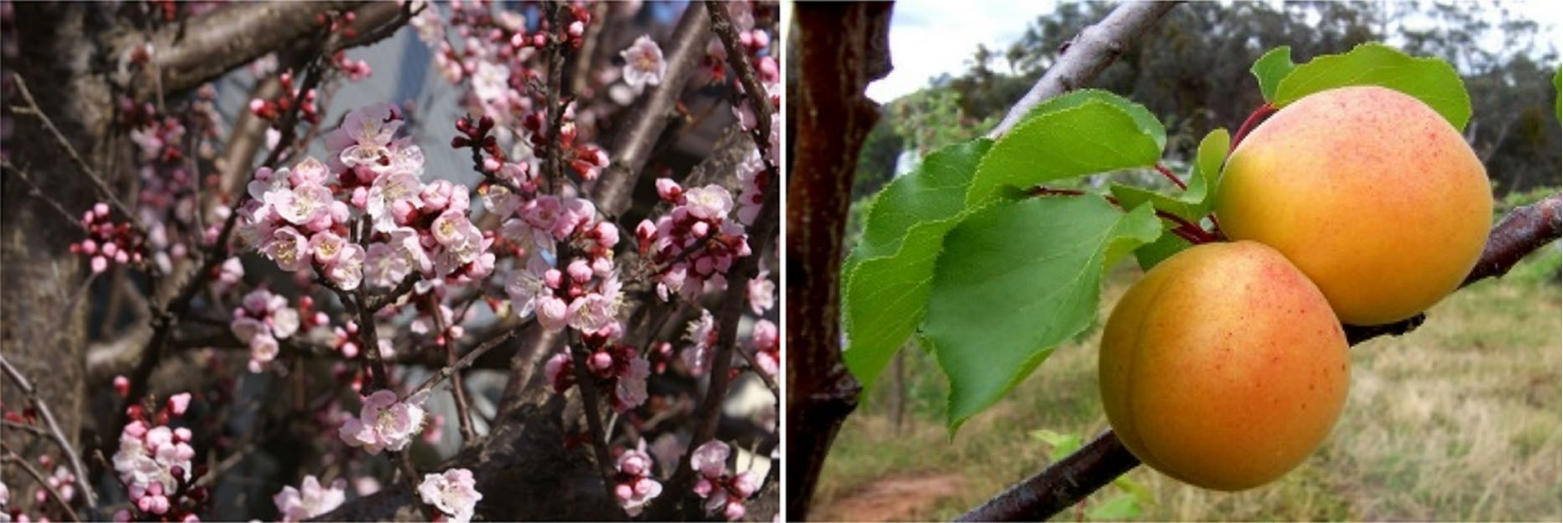
*Prunus armeniaca* L.

**Fig. 4:**
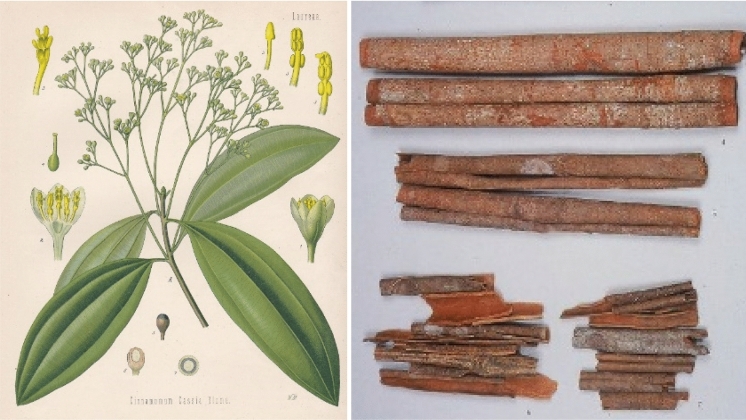
*Cinnamomum cassia* (L.) J.Presl

**Fig. 5:**
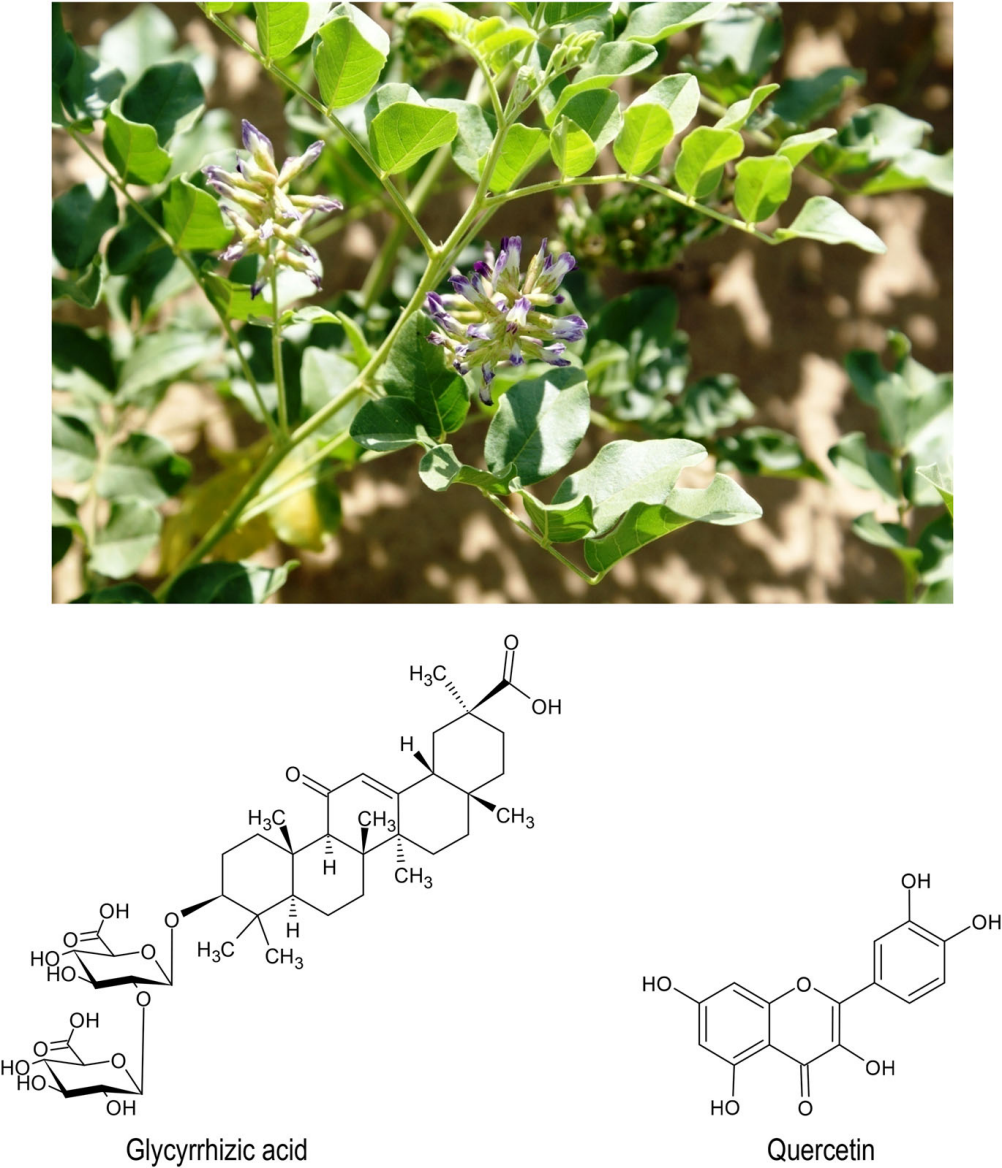
*Glycyrrhiza uralensis* Fisch. ex DC. and some of its active constituents

As mentioned above, Maoto consists of the four individual raw drugs: Ephedrae herba 4 g (Fig. 2), Armeniacae semen 4 g (Fig. 3), Cinnamomi cortex 3 g (Fig. 4), and Glycyrrhizae radix 2 g (Fig. 5). When comparing the composition of this prescription with the other Kampo prescriptions with proven anti-viral effects listed in the above table, three individual raw drugs seem most characteristic for this anti-viral indication, namely Ephedrae herba, Cinnamomi cortex, Glycyrrhizae radix.

Although ephedrine alkaloids in *Ephedra* spp. (Fig. 2) are sometimes regarded as the most important component in Maoto, when ephedrine was removed from *Ephedra* extract, typical side effect like excitement, sleep disorder, palpitations and gastrointestinal disorders could be eliminated in a mouse model (Takemoto et al. 2018). Nevertheless, the same Ephedrine alkaloids-free Ephedra extract (EFE) reduced formalin-induced pain in a dose-dependent manner in male ICR mice that were orally administered 350 mg/kg EFE, or 700 mg/kg Ephedra Herb extract for 3 days (Hyuga et al. 2016). EFE showed anti-influenza virus activity inhibiting the infection of MDCK cells incubated for 72 h in a twofold serial dilution of 10 µM oseltamivir, 50 µg/ml EFE, or 50 µg/ml Ephedra Herb extract with influenza virus A/WSN/33(H1N1) in a concentration-dependent manner (Hyuga et al. 2016). The authors therefore propose an active fraction of the condensed tannin mixture having molecular weight of 45,000 as an alternative active principle of *Ephedra* extract (Takemoto et al. 2018). These finding are especially interesting with regards to the Central Asian species *Ephedra przewalskii* Stapf—also used in the same traditional indications by the natives of both the Western Chinese region of Xinjiang and the Gobi desert in (Inner-) Mongolia—that has been experimentally demonstrated not to contain any significant amounts of ephedrine (Long et al. 2005).

Nevertheless, a study (Wei et al. 2019) aiming to screen antiviral components of the common Ephedrae herba drug confirmed the activity of l-methylephedrin (LMEP), l-ephedrine (LEP) and d-pseudo-ephedrine (DPEP) in MDCK cells infected by mouse-adapted influenza virus A/PR8/34 (H1N1). After 24 h treatment, the virus load in the LMEP 31.25 μg/ml, LEP 15.63 μg/ml and DPEP 15.63 μg/ml groups was significantly lower than that in oseltamivir positive control. In a male ICR mouse model, the mice were treated by gavage with oseltamivir (22 mg/kg), LEP or DPEP (40, 20, 10 mg/kg) solubilized in physiological saline for 7 days, resulting in a significant inhibition of mRNA expression levels of the TLR3, TLR4 and TLR7 signaling pathways and further down-regulated TNF-α levels and up-regulated IFN-β levels (Wei et al. 2019). These Ephedra alkaloids therefore exert an antiviral effect in vitro which may be closely related to the inhibition of viral replication and the modulation of inflammatory response by adjusting the host’s TLRs and RIG-1 (Retinoic acid Inducible Gene I)—an intracellular receptor of the innate immune system—pathways. Further, a vital fluorescence microscopic study (Mantani et al. 1999) showed that the extract of Ephedrae herba (100–400 µg/ml) inhibited the acidification of endosomes and lysosomes in Madin-Darby canine kidney cells in a concentration-dependent manner, inhibiting the growth of influenza A/PR/8/34 (H1N1) (PR8) virus. Conversely, virus growth resumed concomitantly with the reappearance of acidified ELS after removal of the extract. The fact that its inhibitory effect was completely or partially reversed by FeCl_3_, a tannin-reactive agent, indicates that tannins form an active fraction of the extract.

Morimoto et al. (1986) reported that Cinnamon bark contains the procyanidin heptamer cinnamtannin A3 that may contribute to the anti-influenza activity of Maoto. For example, Zhuang et al. (2009) found that the butanol fraction (Fr.2) of Cinnamomi Cortex extract (CC) showed the highest activities of both CC and Fr.2 on wild-type severe acute respiratory syndrome coronavirus (wtSARS-CoV) when the viruses were treated by the extracts before challenging with IC50 values of 43.1 ± 2.8 and 7.8 ± 0.3 µg/ml and SI values of 8.4 and 23.1, respectively. Zhuang et al. (2009) were furthermore able to demonstrate that this extract could interfere with the clathrin-dependent endocytosis pathway using transferrin receptor (TfR) on Jurkat cells as an indicator.

Finally, in the case of Glycyrrhizae radix, the triterpene Glycyrrhizin (or glycyrrhizic acid or glycyrrhizinic acid) (GA) (Fig. 5) has been identified as the principal bioactive ingredient with regards to its anti-viral, anti-inflammatory and hepatoprotective effects. Its anti-viral effects are manifold and have filled an entire review article already (Sun et al. 2019). For example, Hsieh C et al. (2012) were able to demonstrate that GA inhibits PI3K/AKT signaling pathway regulated viral entry, via its neuraminidase inhibiting activity. Utsonomiya et al. (1997) investigated the anti-influenza effect of GA in influenza virus A2 (H2N2) infected BALB/c mice. The results demonstrated that GA may protect mice exposed to a lethal amount of influenza virus by the stimulation of INF-γ production by T cells. The test compound, which consisted of one molecule of GA and two molecules of glucuronic acid was administered intraperitoneally (10 mg per kg of body weight) 1 day before infection and 1 and 4 days post-infection. All of the mice survived over the 21-day experimental period (Utsonomiya et al. 1997). In an in vitro herpes simplex virus-1 (HSV-1) infection model, (Lee et al. 2017) demonstrated that guercetin, a major component of *Glycyrrhiza uralensis*, significantly lowered HSV infectivity in Raw 264.7 cells, resulting in a dramatic decrease in plaque formation in Vero cells when they were incubated with infected cell lysates treated with quercetin. The same concentrations of quercetin further inhibited the expressions of HSV proteins (gD, ICP0) and genes (ICP0, UL13, UL52). Interestingly, quercetin in all tested concentrations specifically suppressed the expression of TLR-3, and this led to the inhibition of inflammatory transcriptional factors (NF-κB and IRF3) (Lee et al. 2017). Moreover, glycyrrhizin in Glycyrrhizae radix is also reported to have anti-influenza activity, as this drug selectively suppressed viral protein synthesis (IC50 = 0.27 mg/ml) in human influenza virus strain A/Udorn/72 (H3N2) on MDCK(+) host cells (Nomura et al. 2019).

Therefore, the anti-influenza activity of Maoto might be accelerated by the addition of the other component herbs besides Ephedrae herba, e.g. Cinnamomi cortex or Glycyrrhizae radix.

With the single exception of Armeniacae semen all the above raw drugs have entered the most recent edition of the European Pharmacopoeia (PhEur 9) as part of an initiative to include East Asian raw drugs in the European regulatory framework in order to facilitate uniform and reliable quality control standards (Table 2).

**Table 2:** 麻黄湯加穿心蓮 (Maoto-ka-senshinren)

Andrographitis herba	4 g
Armeniacae semen	4 g
Cinnamomi cortex	3 g
Ephedrae herba	4 g
Glycyrrhizae radix	2 g

The combination of Maoto and Andrographitis herba is referred to as Ma-Huang-Tang-Jia-Chuan-Xin-Lian in Chinese and in Korean as Mahwang-tang-ga-cheonsimryeon (마황탕가천심련), respectively.

Another very interesting Kampo prescription is Kakkonto (葛根湯), which is listed with the official indication "influenza virus". This prescription is relatively similar to Maoto, consisting of the raw drugs Puerariae radix, Ephedrae herba, Paeoniae radix, Jujubae fructus, Cinnamomi cortex, Glycyrrhizae radix, and Zingiberis rhizoma (Table 1). Especially Paeoniae radix contains gallotannin (Nishizawa et al. 1984) and may therefore be another very promising candidate for the treatment of the early corona virus (COVID-19) infection. This should be especially true for its combination with Andrographitis herba (Table 3).

**Table 3:** 葛根湯加穿心蓮 (Kakkonto-ka-senshinren)

Andrographitis herba	4 g
Cinnamomi cortex	3 g
Ephedrae herba	4 g
Glycyrrhizae radix	2 g
Jujubae fructus	4 g
Paeoniae radix	3 g
Puerariae radix	8 g
Zingiberis rhizoma	1 g

In Chinese, this prescription is referred to as Ge-Gen-Tang-Jia-Chuan-Xin-Lian and in Korean as Galgeun-tang-ga-cheonsimryeon (갈근탕가천심련), respectively.

In the Kampo theory, viral infections as well as infections with bacteria and parasites are all subsumed under the concept "external noxae" (Jap. Gaija/外邪), the traditional indication of both Kakkonto and Maoto. Kakkonto is rather used in cases with sweating and fever, whilst Maoto is used in patients with dry fever and cough. Thus, Kakkonto should also be suitable for the treatment of COVID-19, especially in earlier stage as the combination of fever (ca. 37 °C or higher) and pain fits its traditional indication.

To prevent the development of symptoms, Hochuekkito (Table 1) can be used. For prevention of pneumonia, Saikatsugekito (柴葛解肌湯) has been proposed, which can be combined with Kakkonto or Shosaikoto-ka-kikyo-sekko (小柴胡湯加桔梗石膏). In the stage of pneumonia, next to Western medical treatment, Seihaito (清肺湯) is an option. For the stage of recovery from pneumonia, also Seihaito or Chikujountanto (竹茹温胆湯) have been proposed. (Composition of minor mentioned prescriptions: Appendix 1).

During the recent COVID-19 outbreak, in China traditional Chinese medicine (TCM) was immediately included in the organized clinical response with great success. More than 3100 TCM staff were dispatched to Hubei province and TCM experts were fully integrated in the whole emergency medicine process. This includes a TCM scheme within the official guideline on diagnosis and treatment of COVID-19 (Anonymous 2020a). According to this TCM expert group tasked by the Chinese government with the fight against COVID-19 in Wuhan, the best traditional prescription for the treatment of the infection is Seihaihaidokuto (清肺排毒湯) or Qing-Fei-Pai-Du-Tang in Chinese. Seihaihaidokuto has been officially promoted as a general prescription in the diagnosis and treatment plan of COVID-19 in China (Anonymous 2020a). This state-approved COVID-19 official formula is however very complex and consists of 21 individual herbal drugs (Table 4).

**Table 4:** 清肺排毒湯 (Seihaihaidokuto)

Agastachis herba	9 g
Alismatis rhizoma	9 g
Armeniacae semen	9 g
Asiasari radix	6 g
Asteris radix et rhizoma	9 g
Atractylodis macrocephalae rhizoma	9 g
Aurantii fructus immaturus	6 g
Belamcandae rhizoma	9 g
Bupleuri radix	16 g
Cinnamomi ramulus	9 g
Citri reticulatae pericarpium	6 g
Dioscoreae rhizoma	12 g
Ephedrae herba	9 g
Farfarae flos	9 g
Glycyrrhizae radix	6 g
Gypsum fibrosum	20 g
Pinelliae rhizoma	9 g
Polypori sclerotium	9 g
Poriae sclerotium	15 g
Scutellariae radix	6 g
Zingiberis rhizoma	9 g

Seihaihaidokuto (清肺排毒湯) was developed by combining older prescriptions and thus includes Makyokansekito (麻杏甘石湯), Goreisan (五苓散), Shosaikoto (小柴胡湯), and Yakanmaoto (射干麻黄湯). Goreisan (五苓散) was included in Seihaihaidokuto (清肺排毒湯) because the COVID-19 infection was shown to cause a burst in cytokine production, leading to swellings, inflammation, and diarrhea. Besides prescriptions that directly counteract the symptoms of the COVID-19 infections, Bofutsushosan (防風通聖散) was also included in order to counteract feelings of fullness of the belly and flatulence that typically accompany therapy with high doses of Ephedrae herba. The Chinese National Administration of Traditional Chinese Medicine reports that until the first week of February 2020, 214 COVID-19 patients in the provinces of Shanxi, Hebei, Heilongjiang and Shaanxi were treated with the combination prescription with overall effective rate ≥ 90%. In a majority of patients (≥ 60%) symptoms were markedly improved and in many other the illness was at least stabilized (Zhao et al. 2020). In a subsequent study, 701 COVID-19 patients received the same treatment, with 130 patients (18.5%) completely cured, disappearance of the characteristic symptoms of COVID-19 such as fever and cough in further 51 patients (7.27%), improvement of said symptoms in 268 patients (38.2%), and stabilization of the state of disease in 212 patients (30.2%), respectively (Anonymous 2020b).

Although most experience with Seihaihaidokuto (清肺排毒湯) currently stems directly from Wuhan, successful treatments with very similar combinations such as the parallel administration of Kakkonto (葛根湯), Shoseiryuto (小青竜湯), Goreisan (五苓散), Bofutsushosan (防風通聖散) have been reported from Japan. (Composition of minor mentioned prescriptions: Appendix 1).

In China—in addition to the abovementioned long established prescriptions—some newer TCM formulations have been used for COVID-19 therapy, many of which were developed during the 2002 Severe acute respiratory syndrome (SARS) epidemic (Liu et al. 2012). Of these innovative prescriptions, Shufeng Jiedu Jiaonang (疏风解毒胶囊)—Sofugedokukono (疏風解毒膠囊) in Japanese—has proven especially effective and has also entered the Chinese national treatment guidelines for COVID-19. In pre-clinical studies immunomodulatory and anti-inflammatory effects have been shown against severe actue respiratory syndrom SARS-CoV2-caused pneumonia (Tao et al. 2020). Clinical studies are on their way (Xia et al. 2020; Chen et al. 2021) (Table 5).

**Table 5:** 疏风解毒胶囊 (Shufeng Jiedu Jiaonang)

Bupleuri radix	8 g
Forsythiae fructus	8 g
Glycyrrhizae radix	4 g
Isatidis radix	8 g
Patriniae herba	8 g
Phragmitis rhizoma	6 g
Polygoni cuspidati rhizoma	10 g
Verbenae herba	8 g

Another TCM prescription that first gained prominence during the 2002 SARS epidemic (Liu et al. 2012) for which clinical data are well established (Zheng et al. 2017) and which has been registered and used successfully for the treatment of COVID-19 in China is Fufang Yuxingcao Heiji (复方魚腥草合剤), whose activity against influenza viruses has also been demonstrated in vitro (Zu et al. 2010) (Table 6).

**Table 6:** 复方魚腥草合剤 (Fufang Yuxingcao Heiji)

Forsythiae fructus	0.6 g
Houttuyniae herba	6 g
Isatidis radix	1.5 g
Lonicerae flos	0.6 g
Scutellariae radix	1.5 g

Further clinical examinations of this preparation for COVID-19 therapy are currently ongoing.

## Conclusion

Maoto has the ability to act at all 3 steps important for viral proliferation: It has been shown to enhance the production of antibodies such as IgG, IgM and IgA against influenza virus (Nagai et al. 2014). Maoto further reduces the virus titer (of H1N1 in A549 cells) as well as the production of viral surface proteins such as M2 and neuraminidase (NP) hence preventing viral entry and release (Masui et al. 2017).

In classical Kampo theory, viral infections as well as infections with bacteria and parasites are all subsumed under the concept "external noxae". Maoto-ka-senshinren, i.e. Maoto complemented by Andrographitis herba—a drug with significant anti-viral activity in its own right—can be recommended for the treatment of those infectious diseases that are characterized by fever.

A development of resistance against Maoto-ka-senshinren is not to be expected, as several thousand individual phytochemical constituents are contained in the full extract mixture the continuous application of which should make it almost impossible for the virus to adapt. Furthermore, as stated above, the Maoto prescription has been in continuous and safe use since the second century AD with no known cases of resistance development.

Therefore, we strongly suggest that the combination might be used for influenza viruses and tested for the new corona virus, SARS-COV2 that is currently spreading throughout the world.

## Appendix 1

See Table 7.

**Table 7:** Minor prescriptions

Saikatsugekito (柴葛解肌湯)		
Bupleuri radix	4 g	
Cinnamomi cortex	2 g	
Ephedrae herba	2.5 g	
Glycyrrhizae radix	1 g	
Gypsum fibrosum	6 g	
Paeoniae radix	2 g	
Pinelliae rhizome	3 g	
Puerariae radix	8 g	
Scutellariae radix	2 g	
Zingiberis rhizome	1 g	
Shosaikoto-ka-kikyo-sekko (小柴胡湯加桔梗石膏)		
Bupleuri radix	6 g	
Ginseng radix	2 g	
Glycyrrhizae radix	2 g	
Gypsum fibrosum	10 g	
Jujubae fructus	2 g	
Pinelliae rhizoma	5 g	
Platycodi radix	3 g	
Scutellariae radix	3 g	
Zingiberis rhizoma	4 g	
Seihaito (清肺湯)		
Angelicae sinensis radix	3 g	
Armeniacae semen	2 g	
Asparagi radix	2 g	
Bambusae caulis in taenias	2 g	
Citri reticulatae pericarpium	2 g	
Fritillariae thunbergii bulbus	2 g	
Gardeniae fructus	2 g	
Glycyrrhizae radix	1 g	
Jujubae fructus	2 g	
Mori radicis cortex	2 g	
Ophiopogonis radix	3 g	
Platycodonis radix	2 g	
Poriae sclerotium	3 g	
Schisandrae fructus	1 g	
Scutellariae radix	2 g	
Zingiberis rhizoma	1 g	
Chikujountanto (竹茹温胆湯)		
Aurantii fructus immaturus	2 g	
Bambusae caulis in taenias	3 g	
Bupleuri radix	3 g	
Citri reticulatae pericarpium	2 g	
Coptidis rhizoma	1 g	
Cyperi rhizoma	2 g	
Ginseng radix	1 g	
Glycyrrhizae radix	1 g	
Ophiopogonis radix	3 g	
Pinelliae rhizoma	5 g	
Platycodi radix	2 g	
Poriae sclerotium	3 g	
Zingiberis rhizoma	1 g	
Makyokansekito (麻杏甘石湯) 中		Makyosekikanto (麻杏石甘湯) 日
Armeniacae semen	9 g	4 g
Ephedrae herba	9 g	4 g
Glycyrrhizae radix	6 g	2 g
Gypsum fibrosum	18 g	10 g
Goreisan (五苓散)		
Alismatis rhizoma	6 g	
Atractylodis macrocephalae rhizoma	4 g	
Cinnamomi cortex	3 g	
Polypori sclerotium	4 g	
Poriae sclerotium	4 g	
Shosaikoto (小柴胡湯)		
Bupleuri radix	6 g	
Ginseng radix	2 g	
Glycyrrhizae radix	2 g	
Jujubae fructus	2 g	
Pinelliae rhizoma	5 g	
Scutellariae radix	3 g	
Zingiberis rhizoma	4 g	
Yakanmaoto (射干麻黄湯)		
Asiasari radix	3 g	
Asteris radix et rhizoma	6 g	
Belamcandae rhizoma	9 g	
Ephedrae herba	9 g	
Farfarae flos	6 g	
Jujubae fructus	3 fruits	
Pinelliae rhizoma	9 g	
Schisandrae fructus	3 g	
Zingiberis rhizoma	9 g	
Bofutsushosan (防風通聖散)		
Angelicae sinensis radix	2 g	
Atractylodis macrocephalae rhizoma	3 g	
Cnidii rhizoma	2 g	
Ephedrae herba	2 g	
Forsythiae fructus	2 g	
Gardeniae fructus	2 g	
Glycyrrhizae radix	2 g	
Gypsum fibrosum	3 g	
Menthae haplocalycis herba	2 g	
Natrii sulfus	2 g	
Paeoniae radix	2 g	
Platycodi radix	2 g	
Rhei rhizoma	2 g	
Saposhnikoviae radix	2 g	
Schizonepetae herba	2 g	
Scutellariae radix	2 g	
Talcum crystallinum	5 g	
Zingiberis rhizoma	2 g	

## Appendix 2

See Table 8.

**Table 8:** Taxonomy of East Asian herbal drugs

Drug	Kanji	Accepted name https://mpns.science.kew.org/mpns-portal/
Agastachis herba	藿香	*Agastache rugosa* (Fisch. & C.A.Mey.) Kuntze
Alismatis rhizoma	澤瀉	*Alisma plantago-aquatica subsp. orientale* (Sam.) Sam
Andrographitis herba	穿心蓮	*Andrographis paniculata* (Burm.f.) Nees
Angelicae sinensis radix	當歸	*Angelica sinensis* (Oliv.) Diels
Armeniacae semen	杏仁	*Prunus armeniaca* L.
Asiasari radix	細辛	*Asarum sieboldii* Miq
Asparagi radix	天門冬	*Asparagus cochinchinensis* (Lour.) Merr
Asteris radix et rhizoma	紫菀	*Aster tataricus* L.f
Astragali radix	黄芪	*Astragalus mongholicus* Bunge
Atractylodis macrocephalae rhizoma	白朮	*Atractylodes macrocephala* Koidz
Aurantii fructus immaturus	枳實	*Citrus* × *aurantium* L.
Bambusae caulis in taenias	竹茹	*Bambusa beecheyana* Munro
Belamcandae rhizoma	射干	*Iris domestica* (L.) Goldblatt & Mabb
Bupleuri radix	柴胡	*Bupleurum falcatum* L. (used in Japan & Korea) *Bupleurum chinense* DC. (used in China)
Cimicifugae rhizoma	升麻	*Actaea dahurica* (Turcz. ex Fisch. & C.A.Mey.) Franch
Cinnamomi cortex	桂皮	*Cinnamomum cassia* (L.) J.Presl
Cinnamomi ramulus	桂枝	*Cinnamomum cassia* (L.) J.Presl
Citri reticulatae pericarpium	陳皮	*Citrus* × *aurantium* L.
Cnidii rhizoma	川芎	*Ligusticum officinale* (Makino) Kitag
Coptidis rhizoma	黄連	*Coptis chinensis* Franch
Cyperi rhizoma	香附	*Cyperus rotundus* L.
Dioscoreae rhizoma	山薬	*Dioscorea japonica* Thunb
Ephedrae herba	麻黄	*Ephedra sinica* Stapf
Farfarae flos	款冬	*Tussilago farfara* L.
Forsythiae fructus	連翹	*Forsythia suspensa* (Thunb.) Vahl
Fritillariae thunbergii bulbus	浙貝母	*Fritillaria thunbergii* Miq
Gardeniae fructus	梔子	*Gardenia jasminoides* J.Ellis
Ginseng radix	人参	*Panax ginseng* C.A.Mey
Glycyrrhizae radix	甘草	*Glycyrrhiza uralensis* Fisch. ex DC
Houttuyniae herba	十薬	*Houttuynia cordata* Thunb
Isatidis radix	藍草	*Isatis indigotica* Fort
Jujubae fructus	大棗	*Ziziphus jujuba* Mill
Lonicerae flos	金銀花	*Lonicera japonica* Thunb
Lycii radicis cortex	地骨皮	*Lycium chinense* Mill
Menthae haplocalycis herba	薄荷	*Mentha canadensis* L.
Mori radicis cortex	桑白皮	*Morus alba* L.
Ophiopogonis radix	麥冬	*Ophiopogon japonicus* (Thunb.) Ker Gawl
Paeoniae radix	芍薬	*Paeonia lactiflora* Pall
Patriniae herba	敗醤葉	*Patrinia scabiosifolia* Fischer ex Treviranus
Phragmitis rhizoma	蘆根	*Phragmites communis* Trin
Pinelliae rhizoma	半夏	*Pinellia ternata* (Thunb.) Makino
Platycodonis radix	桔梗	*Platycodon grandiflorus* (Jacq.) A.DC
Polygoni cuspidati rhizoma	虎杖根	*Polygonum cuspidatum* Sieb.et Zucc
Polypori sclerotium	豬苓	*Polyporus umbellatus* (Pers.) Fries
Poriae sclerotium	茯苓	*Poria cocos* (Schw.) Wolf
Puerariae radix	葛根	*Pueraria montana var. lobata* (Willd.) Maesen & S.M.Almeida ex Sanjappa & Predeep
Rhei rhizoma	大黄	*Rheum palmatum* L.
Saposhnikoviae radix	防風	*Saposhnikovia divaricata* (Turcz. ex Ledeb.) Schischk
Schisandrae fructus	五味子	*Schisandra chinensis* (Turcz.) Baill
Schizonepetae herba	荊芥	*Nepeta tenuifolia* Benth
Scutellariae radix	黄芩	*Scutellaria baicalensis* Georgi
Verbenae herba	馬鞭草	*Verbena officinalis* L.
Zingiberis rhizoma	生姜	*Zingiber officinale* Roscoe
